# Biological Characterization and Evolution of Bacteriophage T7-△holin During the Serial Passage Process

**DOI:** 10.3389/fmicb.2021.705310

**Published:** 2021-08-02

**Authors:** Hai Xu, Xi Bao, Weiming Hong, Anping Wang, Kaimin Wang, Hongyan Dong, Jibo Hou, Roshini Govinden, Bihua Deng, Hafizah Y. Chenia

**Affiliations:** ^1^Jiangsu Key Laboratory for High-Tech Research and Development of Veterinary Biopharmaceuticals, Jiangsu Agri-Animal Husbandry Vocational College, Taizhou, China; ^2^Institute of Veterinary Immunology & Engineering, Jiangsu Academy of Agricultural Science, Nanjing, China; ^3^School of Life Sciences, College of Agriculture, Engineering and Science, University of KwaZulu-Natal, Durban, South Africa; ^4^Animal, Plant and Food Test Center of Nanjing Customs, Nanjing, China; ^5^Jiangsu Co-Innovation Center for Prevention and Control of Important Animal Infectious Diseases and Zoonoses, Yangzhou, China

**Keywords:** T7 phage, holin, lysis, compensatory host, evolution

## Abstract

Bacteriophage T7 gene *17.5* coding for the only known holin is one of the components of its lysis system, but the holin activity in T7 is more complex than a single gene, and evidence points to the existence of additional T7 genes with holin activity. In this study, a T7 phage with a gene *17.5* deletion (T7-△holin) was rescued and its biological characteristics and effect on cell lysis were determined. Furthermore, the genomic evolution of mutant phage T7-△holin during serial passage was assessed by whole-genome sequencing analysis. It was observed that deletion of gene *17.5* from phage T7 delays lysis time and enlarges the phage burst size; however, this biological characteristic recovered to normal lysis levels during serial passage. Scanning electron microscopy showed that the two opposite ends of *E. coli* BL21 cells swell post-T7-△holin infection rather than drilling holes on cell membrane when compared with T7 wild-type infection. No visible progeny phage particle accumulation was observed inside the *E. coli* BL21 cells by transmission electron microscopy. Following serial passage of T7-△holin from the 1st to 20th generations, the mRNA levels of gene *3.5* and gene *19.5* were upregulated and several mutation sites were discovered, especially two missense mutations in gene *19.5*, which indicate a potential contribution to the evolution of the T7-△holin. Although the burst size of T7-△holin increased, high titer cultivation of T7-△holin was not achieved by optimizing the culture process. Accordingly, these results suggest that gene *19.5* is a potential lysis-related component that needs to be studied further and that the T7-△holin strain with its gene *17.5* deletion is not adequate to establish the high-titer phage cultivation process.

## Introduction

Virulent T7 phage is one of seven phages first identified in *Escherichia coli* in 1945 (Demerec and Fano, 1945). The genome of T7 phages is ∼40 kb and is packed into the polyhedral head formed by the capsid proteins. The capsid is composed predominantly of the products of gene *10*, which encodes two kinds of protein, i.e., gp10A (344 aa) and gp10B (397 aa). Due to a reading frame shift at the end of gene *10*, gp10A and gp10B are expressed at the ratio of 9:1 under normal culture conditions (Sipley et al., 1991). However, the proportion of gp10A and gp10B may vary according to conditions and does not affect the integrity of phage particles (Condron et al., 1991; Deng et al., 2018). Based on the common principle of phage display, exogenous peptides are fused with a phage capsid protein, which allows them to be displayed on the surface of the phage particle. Thus, T7 phages have been employed in a phage surface display system (Rosenberg et al., 1996) and commercialized by Novagen.

Since George Smith first established the phage display system in 1985, numerous bacteriophage species have been engineered for phage display, including M13, f1, T4, and phage λ (Zambrano-Mila et al., 2020). Phage display technology is beneficial to several biological fields due to its powerful mechanism for production of high copy numbers of peptides or proteins, the analysis of protein interactions (Pande et al., 2010), the isolation of functional compounds, and the study of antigen–antibody binding (Hamzeh-Mivehroud et al., 2013). Various new applications in the biotechnology industry include phages playing a key role as delivery vehicles for proteins and DNA vaccines, as gene therapy delivery vehicles, and as detection tools for pathogenic organisms (Clark and March, 2006). These applications may require large quantities of phage particles, but the infection and reproduction rates are low, which is often the case in an engineered phage (Krysiak-Baltyn et al., 2018). Consequently, there is an increased demand for high-titer or large-scale phage culture technology.

Cost-effective and scalable methods for phage production are thus required. Previous studies have established computational models to assist the optimization of phage production processes, especially in the context of large-scale phage production (Krysiak-Baltyn et al., 2018). Moreover, the timing of lysis is an important phage trait that directly determines the burst size. A short lysis duration of phage-infected cells results in the drawback of releasing few progeny particles, while a prolonged lysis time allows the phage to utilize the host bacteria more efficiently, producing more progeny particles. The two-component genetic model of lysis is essential to most large double-stranded phages, although the specific control mechanisms vary (Wang et al., 2000; Xu et al., 2004, 2005). This two-component model involves a phage-encoded endolysin and holin. Furthermore, it has also been demonstrated that spanin, a third component, plays a key role in the final step of host lysis (Rajaure et al., 2015). The holin is a small membrane protein that accumulates in the inner membrane and suddenly forms holes and has been proposed as a clock to control the timing of lysis (Young, 1992). The only known holin in T7 phage is encoded by gene *17.5*; however, gene *17.5* is non-essential for T7 phage, which suggests the possibility of additional holin genes being present (Heineman and Bull, 2007). This then suggests the potential of regulating holin to indirectly control the lysis time in order to achieve the goal of high-titer cultivation.

In this study, a gene *17.5* (holin) deletion T7 phage was constructed and serially passaged for 20 generations to evaluate the evolution of biological characteristics during this process. We verified that the holin gene is non-essential for T7 life cycle but delayed the lysis time, corroborating a previous study (Heineman et al., 2009). Our current study results demonstrate for the first time the evolution of a T7-△holin phage during the serial passage process. It was observed that the biological characteristics of the T7-△holin phage gradually tended toward the wild-type (wt) T7 phage during the continuous passage process. Moreover, whole-genome sequencing (WGS) analysis identified several mutations distributed in the T7-△holin phage genome, which could not be correlated with the phage biological characteristics. An upregulation of mRNA levels was observed in the T7-△holin phage, especially genes that directly or potentially participate in the lysis process.

## Materials and Methods

### Bacteria and Phage Preparation

**FIGURE 1:**
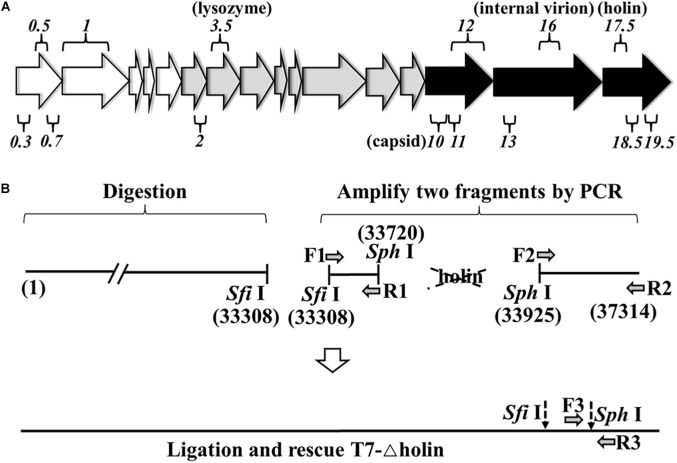
Rescue of T7-△holin mutant phage. **(A)** Major transcripts from bacteriophage T7 genome. Empty arrows on the left represent early genes, gray arrows in the middle represent intermediate genes, and black arrows on the right represent late genes. Among the 56 known or potential genes, only a selected subset of genes mentioned in the text is shown. The numbers over and under the arrows indicate gene designation. **(B)** Strategy used to rescue a holin gene deletion mutant phage. The number in bracket stands for the gene position. The dotted arrows indicate the restriction enzyme sites.

T7 select 415-1b phage was purchased from Merck KGaA (Darmstadt, Germany). T7 select 415-1b phage (referred to as T7-wt) has the same skeleton sequence (Figure 1A) with the original T7 phage (GenBank accession number V01146; Dunn and Studier, 1983) except for two main deletions at sites 579-2717 and 11163-11515 (Nucleotide numbers are those of original T7 phage) and a multiple cloning site construction at the end of *p10A* gene. *E. coli* BL21 was used as the host organism for T7 phage infection and cultivation. Phage lysate was prepared by adding phage stock with a multiplicity of infection (MOI) = 1:1,000 into the *E. coli* BL21 host culture with an *OD*_600 *nm*_ of ∼1.0 and shaking until the culture was clarified. Phage titer was measured using a previously described double agar overlay plaque assay (Kropinski et al., 2009). Briefly, 100 μl of a dilution of the phage sample was added to 200 μl of a bacterial suspension and incubated at room temperature for 3 min. The mixture was added to 4 ml top agar, gently homogenized, and poured into a 100-mm Petri dish previously prepared with 10 ml of bottom agar. Plates were gently swirled and dried for 5 min and incubated at 37°C for 4 h. *E. coli* BL21-DE3 carrying pET-32a containing the gene *17.5* insert (BL-holin host) was used as the compensatory host for holin deletion phage.

### Construction of Holin Deletion Phage

For the construction of T7-△holin phage, a gene *17.5* deletion T7 phage was constructed and rescued (Figure 1B) using a previously published strategy (Chan et al., 2005). Briefly, T7-wt phage linear genome was extracted (Pickard, 2009; Jakočiūnė and Moodley, 2018) and digested using *Sfi*I, and the upstream fragments from the single-cut site were gel extracted. The holin deletion fragment was prepared using a PCR-based protocol. A holin upstream part (from *Sfi*I site to T7-wt genomic position 33720) and a holin downstream part (from T7-wt genomic position 33925 to the right terminal position 37314) were PCR amplified using F1/R1, F2/R2 primers, respectively. Moreover, a *Sph*I restriction enzyme site was introduced by primer. The holin deletion genome was ligated using two single restriction sites, *Sfi*I and *Sph*I. The ligation mixture was then combined with T7 phage packaging extract to rescue infective phage particles. Primers F3/R3 were designed by choosing the 300 bp upstream and downstream of the *Sph*I site, and a plaque PCR method was used to identify the correct mutant phage clone. PCR amplimers equivalent to 600 bp were regarded as the correct mutant phage clone and named T7-△holin. All PCR primers that were used in this study are listed in Supplementary Table 1.

### Electron Microscopy

For SEM and TEM detection, *E. coli* BL21 cells were grown to a density of 10^9^ CFU/ml and infected with T7-△holin and T7-wt phage stocks at a MOI of 10. Uninfected cells were used as a control. Samples were taken at 20 min post-infection and fixed with 3% (vol/vol) glutaraldehyde. After 30 min of fixation at room temperature, cells were collected, washed twice with 10 mM potassium phosphate buffer (pH 7.2), and prepared for electron microscopy as described previously (Bamford and Mindich, 1980). Samples were sent to the Testing & Analysis Center at Yangzhou University for SEM and TEM analyses.

### Serial Passage of T7-△holin for Experimental Evolution

Serial passage was conducted by adding T7-△holin phage to 20 ml of log-phase *E. coli* BL21 host cells (*OD*_600 *nm*_ of 0.5–1.0) at a low MOI (0.01). The culture was shaken at 37°C until complete lysis of the host cells was obtained. A drop from the previous passage was then added to another 20 ml of a new log-phase *E. coli* BL21 host culture, and the process was repeated 20 times. If the phage lysate was kept overnight before the next passage, 50 μl of chloroform was added and the mixture was stored at 4°C.

### Measurement of Burst Size

Phage burst sizes were measured as previously described (Heineman et al., 2009) with some modification. Briefly, *E. coli* BL21 cells were agitated until the *OD*_600 *nm*_ reached 0.3–0.5 and then infected with phages at a MOI of 0.01. When using a BL-holin host, 1 mM isopropyl β-D-1-thiogalactopyranoside (IPTG) was added when the *OD*_600 *nm*_ reached 0.2 and agitation was continued for 30 min. After allowing phages to be adsorbed to host cells for 2 min, cells were centrifuged to discard all the supernatant and immediately diluted 10,000-fold into pre-warmed Luria-Bertani (LB) media, followed by further incubation for 30–35 min. Samples were extracted every 5 min, and a 10^3^ dilution was used for titer measurements made early in the assay while subsequent assays used a 10^6^ dilution in order to more effectively prevent further adsorption. Comparisons between titers with and without chloroform at 5 and 10 min after infection were used to determine the number of unabsorbed phages and thus the number of infected cells. Burst sizes were calculated as the number of phages released per infected cell, and the data from three independent experiments were assessed with a sigmoidal curve model using the least-squares fitting method (SPSS, version 2.0).

### Kinetic Assay of Lysis

The kinetics of lysis was assayed as previously described with some modification (Nguyen and Kang, 2014). The *E. coli* BL21 host was cultured with agitation until an *OD*_600 *nm*_ of 0.3 was reached and then infected with phages at a high MOI of 3–4. Adsorption was allowed to proceed for 2 min, and the culture was centrifuged to discard all free phages. The pellet was resuspended in fresh LB medium and allowed to grow at 37°C in a shaking incubator. At every 5 min after infection, 200 μl of the culture was removed and placed into wells of a 96-well plate kept on ice. Cell density was measured at *OD*_600 *nm*_ after all samples were taken. A lysis curve and average lysis time were estimated by fitting the data from at least two independent experiments.

### Phage Adsorption Assay

The adsorption rates of phage to the *E. coli* BL21 host was measured as previously described (Nguyen and Kang, 2014). Fresh host cells were prepared with agitation until the logarithmic growth phase was reached and diluted in pre-warmed LB medium to a final concentration of 1 × 10^7^ CFU/ml. *E. coli* BL21 host cells (5 ml) were pre-warmed at 37°C for 10 min and mixed with phage solution to a final concentration of 1 × 10^5^ PFU/ml in SM buffer. After 5 min of infection at 37°C, 200 μl of mixture was sampled and centrifuged immediately to separate free phages and adsorbed phages. Meanwhile, another 200 μl of mixture was sampled and kept uncentrifuged. The phage titer of both centrifuged (N_*f*_) and uncentrifuged (N_*t*_) phages was determined by the plaque assay using the *E. coli* BL21 host. The adsorption rate, α, was calculated using the equation α = –0.2 [ln (N_*f*_/N_*t*_)] (Nguyen and Kang, 2014).

### Quantification of Phage mRNA Levels

The mRNA levels of phage genes (gene *3.5*, *10a*, *16*, *17*, *18.5*, and *19.5*) in infected host cells were measured quantitatively by reverse transcription followed by real-time quantitative PCR (RT-qPCR) method (Nguyen and Kang, 2014). Total RNA was extracted using RNeasy Protect Bacteria Mini Kit (Qiagen, Hilden, Germany) from phage-infected *E. coli* BL21 host (15 min post-infection) and reverse transcribed using 15-mer random primers (Takara Bio Inc., Shiga, Japan). Quantitative PCR for target mRNAs was performed using a CFX Connet system (Bio-Rad, CA, United States). *E. coli* BL21 host 16S rRNA was used as a reference control. The primer details are presented in Supplementary Table 1. All data were analyzed statistically using the two-tailed Student’s *t*-test (Clokie, 2009).

### Whole-Genome Sequencing

Genomic DNA for WGS analysis was extracted from the 1st, 10th, and 20th generations of T7-△holin phage obtained following the serial passage process using the phenol chloroform technique with minor modifications (Pickard, 2009; Jakočiūnė and Moodley, 2018). This involved the whole phage population, which was collected from the respective serial passage generation. Sequencing samples were prepared with the Nextera XT DNA library preparation kit (Illumina, San Diego, CA, United States). The normalized library was sequenced on a MiSeq platform (Illumina, San Diego, CA, United States) with a 300-cycle MiSeq Reagent Micro Kit V2 (Illumina, San Diego, CA, United States). Raw sequencing data were subjected to data cleaning by removing adapters, trimming low-quality ends, depleting sequences with lengths of less than 36 nt, and sequencing quality analysis with FastQC. The taxonomy of cleaned reads was classified using Kraken v0.10.5-beta. Reads of the T7 phage were extracted from the Kraken classification results and *de novo* assembled with SPAdes (v 3.5.0).

### High-Titer Phage Production

The high-titer phage production process was conducted by the optimization of MOI and host cell density. *E. coli* BL21 was cultured in 200-ml LB medium by agitation at 250 rpm and 37°C, and 10-ml aliquots were taken when the *OD*_600 *nm*_ reached 0.5, 1.0, 1.5, respectively. T7-△holin stocks of different generations were prepared and used to infect *E. coli* BL21 at different MOIs (0.01, 0.001, 0.0001). T7-wt phage was used as a control. The mixed phage–host culture was further agitated at 250 rpm and 37°C until all the host cells were completely lysed. Culture supernatant was collected by centrifugation, and phage titers were determined by the double-layer agar method (Kropinski et al., 2009). The test was repeated three times for each sample, and the mean value was recorded as the final phage titer. The total amount of capsid protein in the lysate was detected by the Dot-ELISA method (Xu et al., 2017), since there was T7 tag attached to the N-terminal of the capsid protein. Briefly, lysate samples that were collected from the cultivation process were serially diluted 10-fold, fixed on nitrocellulose membrane, and probed with horseradish peroxidase (HRP)-labeled anti-T7 tagged monoclonal antibody (1:10,000 dilution) (Merck). Visualization was carried out using an HRP and a diaminobenzidine tetrahydrochloride (DAB) chromogenic development kit (Beyotime, China).

## Results

### Generating a T7 Phage Mutant by Deleting the Holin Gene

A T7-△holin phage was created by deleting gene *17.5* to evaluate the roles of holin in the T7 phage lytic cycle. As holin is a later expressed gene and is located downstream in the linear genome (Figure 1A), a 204-bp DNA fragment was deleted from the T7-wt genome. T7-wt genome was digested by *Sfi*I, and the upstream fragments from the restriction site were gel extracted (Supplementary Figure 1). Two gene fragments were prepared by PCR, and a *Sph*I site was introduced using the primer (Supplementary Figure 2). Three gene fragments were ligated through the *Sfi*I and *Sph*I site and after being rescued named the T7-△holin phage (Figure 1B). Deletion of the holin gene was confirmed by plaque PCR detection and DNA sequencing analysis. Accordingly, gene *17.5* deletion T7 phage was rescued using *E. coli* BL21-ED3 carrying a pET-32a containing gene *17.5* insert (BL-holin host) and confirmed.

### Electron Microscopy of Host Cells Infected With Phage

**FIGURE 2:**
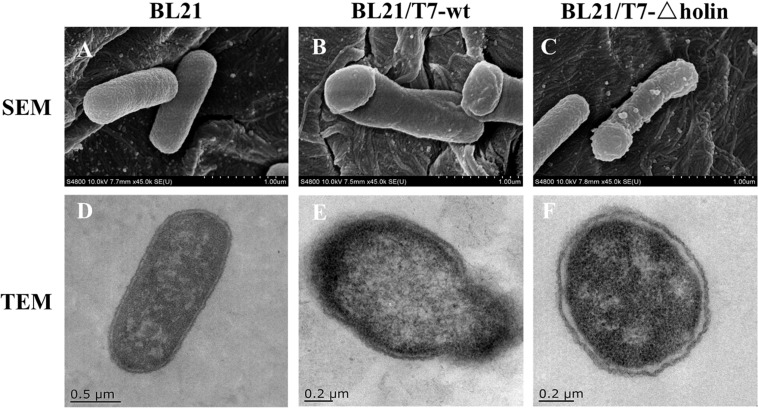
Electron microscopy observation of *Escherichia coli* BL21 cells infected with T7 phage. **(A)** SEM of *Escherichia coli* BL21 cell control. **(B)** SEM of *E. coli* BL21 cell infected with T7-wt. **(C)** SEM of *E. coli* BL21 cell infected with T7-holin. **(D)** TEM of *E. coli* BL21 cell control. **(E)** TEM of *E. coli* BL21 cell infected with T7-wt. **(F)** TEM of *E. coli* BL21 cell infected with T7-holin.

In order to elucidate the injury of the host cell membrane after phage infection, both SEM and TEM analyses were conducted. As anticipated, uninfected *E. coli* BL21 control cells maintained their intact shape (Figures 2A,D), while obvious cell injury was observed in phage-infected cells. Slight differences were observed in membrane lesions following T7-wt and T7-△holin infections. After T7-wt infection, the lysis event was initiated from the pole of the cells as exhibited by indentation of the bacterial cell (Figure 2B). Visible pores appeared on the cell surface, which were partially collapsed by releasing cytoplasmic contents and escape of newly packaged offspring phage particles (Figure 2E). In contrast, after 20 min of T7-△holin infection, bacterial cells exhibited swelling at the poles but still maintained their intact structure (Figure 2C). Subtle interval enlargement was observed between the inner and outer membrane, and suspected phage particles were observed in the cytoplasm (Figure 2F). These results are consistent with a previous finding that T7-wt phage holins form lesions in the host cell inner membrane (Dewey et al., 2010). The shape change of T7-△holin-infected cells indicates that an unknown compensation mechanism exists that is different from T7-wt infection.

### Phenotypic Characterization of T7-△holin Phage

**FIGURE 3:**
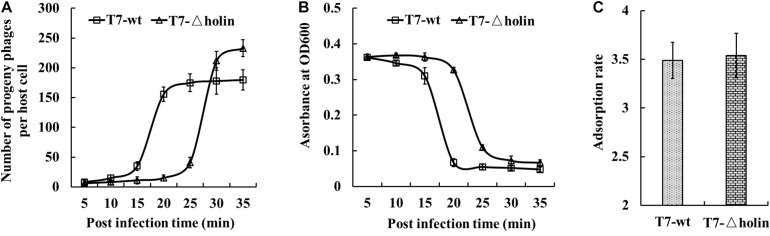
Phenotypic characterization of T7-△holin deletion phage. **(A)** One-step growth curve for burst size measurement of the T7-wt (squares) and T7-△holin (triangles) phages. **(B)** Lysis curves for estimation of onset of lysis for T7-wt and T7-△holin phage infections. **(C)** Adsorption rates measured for the T7-wt and T7-△holin phages. Vertical error bars represent standard deviations.

The 1st, 10th, and 20th generation stocks were prepared, and their phenotypic effects in both *E. coli* BL21 and BL-holin hosts were assessed (Table 1). With respect to the life cycle of a lytic phage, the burst size, lysis timing, and adsorption rate are important indices of phenotype (Nguyen and Kang, 2014). When measured under the conditions where the *E. coli* BL21 host was in an early log phase, the burst sizes of T7-wt and T7-△holin phages were 182 ± 5.3 and 221 ± 5.7 (*p* = 0.002) particles per host, respectively (Figure 3A and Table 1). The T7-△holin phage achieved a burst size 121% that of the burst size of the T7-wt phage (*p* = 0.002); however, only 175 progeny phage particles were produced when the host bacterium was switched to the *E. coli* BL-holin strain. Lysis was delayed by approximately 15 min for the T7-△holin phage compared to the T7-wt phage when the *E. coli* BL21 host was infected (Figure 3B and Table 1). Lysis was also accelerated when T7-△holin phage infected the *E. coli* BL-holin host. The optimal lysis time correlated positively with adsorption rate, and it has been observed previously that phage strains with higher adsorption rates show more rapid lysis (Shao and Wang, 2008). No significant difference in the adsorption rates of T7-wt and T7-△holin (Figure 3C and Table 1) was observed (*p* = 0.773). Thus, deletion of holin gene from T7 phage genome led to a delay in lysis time and enlargement of burst size; however, it had no effect upon the phage adsorption to the host bacteria (Table 1).

**TABLE 1:** Phenotypic characterization of T7-△holin phage infection of *Escherichia coli* BL21 cells.

**Phage/Host**	**Burst size (phage/cell)**	**Lysis time (min)**	**Adsorption rate (phage/ml/min)**
T7-wt/BL21	182 ± 5.3	15.2 ± 1.6	3.8 ± 0.4
T7-△holin-F1/BL21	221 ± 5.7 (*p* = 0.002)	29.3 ± 4.5 (*p* = 0.013)	3.7 ± 0.3 (*p* = 0.773)
T7-△holin-F1/BL-holin	175 ± 9.4 (*p* = 0.456)	13.3 ± 1.9 (*p* = 0.398)	3.5 ± 0.2 (*p* = 0.688)
T7-△holin-F10/BL21	214 ± 9.5 (*p* = 0.014)	28.6 ± 2.1 (*p* = 0.001)	3.9 ± 0.4 (*p* = 0.291)
T7-△holin-F10/BL-holin	175 ± 8.0 (*p* = 0.119)	14.3 ± 3.2 (*p* = 0.124)	3.7 ± 0.5 (*p* = 0.115)
T7-△holin-F20/BL21	193 ± 9.1 (*p* = 0.103)	23.7 ± 2.5 (*p* = 0.033)	3.7 ± 0.5 (*p* = 0.952)
T7-△holin-F20/BL-holin	174 ± 7.1 (*p* = 0.742)	13.6 ± 2.4 (*p* = 0.830)	3.4 ± 0.3 (*p* = 0.531)

*T7-△holin-F1, T7-△holin-F10, T7-△holin-F20 stands for the 1st, 10th, and 20th generation of T7-△holin phage, respectively. BL-holin stands for the compensatory host carrying a pET-32a expression holin protein. “*p*” values were calculated against the T7-wt.*

### Serial Passage and Plaque Enlargement Assay

To examine the emergence of adaptive changes induced by passage of T7-△holin phage, the mutant strain was serially passaged 20 times in the *E. coli* BL21 host. It was hypothesized that after serial passages in the *E. coli* BL21 host, T7-△holin would gradually adapt to growth without the holin gene, thereby leading to phenotypic and/or genotype changes. As expected, there was burst size shrinkage of T7-△holin after serial passage from 10th generation (214 particles per cell) to the 20th generation (193 particles per cell) in *E. coli* BL21 host, and lysis accelerated from 28.6 min to 23.7 min (Table 1). However, both the burst size and lysis time of T7-△holin (F1, F10, and F20 generation) were not significantly different in the *E. coli* BL-holin host (Table 1).

**FIGURE 4:**
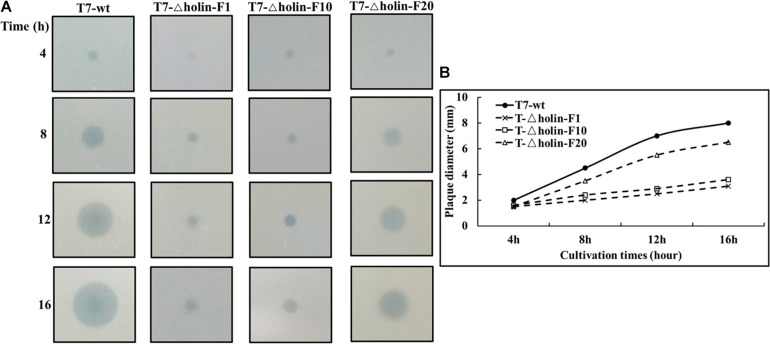
Plaque enlargement assay of T7-wt and T7-△holin. **(A)** Images of T7-wt and different generations of T7-△holin phage plaques formed on a lawn of *Escherichia coli* BL21 host cells over a 16-h incubation period. **(B)** The plaque diameter of T7-wt and T7-△holin phages formed on a lawn of *E. coli* BL21 host cells over a 16-h incubation period.

A phage plaque is a clearing in a bacterial lawn, and plaques form *via* an outward diffusion of phage progeny that preys on surrounding bacteria (Tabib-Salazar et al., 2018). So, the size of phage plaque can serve as an index as to how efficiently a phage developed and produced progeny within an infected bacterial host. As shown in Figure 4A, the plaque size on a lawn of T7-△holin-F1 was barely distinguishable for the first 4 h, in contrast to T7-wt, T7-△holin-F10, and T7-△holin-F20, which had visible plaques on the host lawn. With an increase in incubation time (8, 12, and 16 h), the plaque of T7-wt and T7-△holin-F20 continued to enlarge, while the plaques of T7-△holin-F1 and T7-△holin-F10 increased more slowly (Figure 4B). This difference in plaque size correlated with burst size and lysis time data shown in Table 1. These data indicate that the T7-△holin mutant gradually adapted to the holin deletion and phenotypically recovered with increasing serial passage.

### mRNA Levels of T7 Capsid, Tail Fiber, and Lysis-Related Proteins

The capsid and tail fibers are two of the major proteins that make up the T7 phage particle. Gene *10a* encodes the primary capsid protein that is the outer protective shell of the phage, and gene *17* encodes the tail fiber that is the key protein interacting with the *E. coli* surface lipopolysaccharide (Lin et al., 2012). Gene *3.5* (lysozyme) and gene *17.5* (holin) comprise the two-component lysis system module of T7 phage, and it has been suggested that genes *16*, *18.5*, and *19.5* are lysis-related genes, with gene *19.5* being compensatory for loss of holin function (Heineman et al., 2009).

**FIGURE 5:**
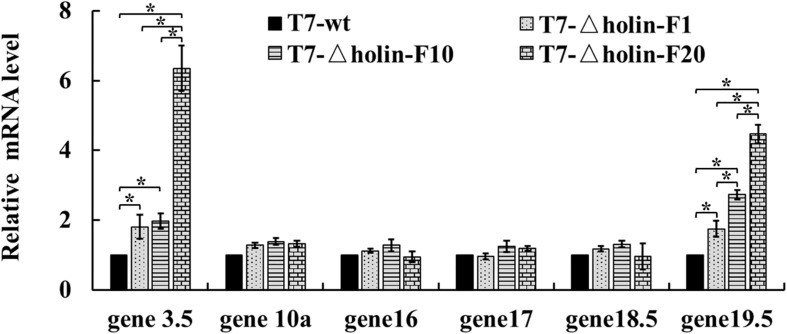
The mRNA levels of T7-wt and T7-△holin during infection. The mRNA levels of several T7 genes in the T7-wt and the 1st, 10th, and 20th generations of the T7-△holin phage infections measured using quantitative real-time PCR. *Escherichia coli* BL21 cell 16S rRNA was set as a reference control. All data were statistically analyzed using the two-tailed Student’s *t*-test. Bars with a * indicate significant difference (*p* < 0.05).

The mRNA levels of these genes (genes *3.5*, *10a*, *16*, *17*, *18.5*, and *19.5*) were quantitatively measured after 10 min of infection using RT-qPCR (Figure 5). The mRNA levels of the major proteins genes *10a* and *17* were not significantly different between the T7-wt and three generations of T7-△holin (*p* > 0.05), as well as those of the two potential lysis-related genes *16* and *18.5*. However, mRNA levels corresponding to genes *3.5* and *19.5* were significantly elevated in the T7-△holin infection. Gene *3.5* mRNA levels increased 1. 8-, 2. 0-, and 6.4-fold for T7-△holin-F1, T7-△holin-F10, and T7-△holin-F20, respectively. Gene *19.5* mRNA levels increased 1. 7-, 2. 7-, and 4.5-fold for T7-△holin-F1, T7-△holin-F10, and T7-△holin-F20, respectively. These data suggested that the upregulated expression of genes *3.5* and *19.5* may compensate for the lack of the holin protein and resulted in a more rapid onset of lysis compared to the T7-△holin-F1 mutant.

### Genetic Evolution During Serial Passage

A WGS approach was undertaken to map potential compensatory mutations (Table 2) and identify genes responsible for the adaptation of T7-△holin during the serial passage process. WGS data of three different generations of T7-△holin (1st, 10th, and 20th) were generated and compared to those of the T7-wt parent strain. Point mutations were distributed throughout the entire genome, and the mutation frequency increased with the serial passage generations. Nonsense mutants predominated and were observed in non-essential genes *1.6*, *1.7*, *2.8*, and *5.3*, as well as essential genes *5*, *15*, *16*, and *19*. Examination of the two lysis-related genes *16* and *19.5* (Table 2) indicated missense mutations from the 1st generation (F1) followed by accumulation of another mutation in the 20th generation (F20). Surprisingly, no mutations arose in the main lysis gene *3.5* (lysozyme) during this passage process. Heineman et al. (2009) have previously described the lytic activity of gene *16* and identified gene *19.5* as a possible gene with lytic function. Since the current sequencing data (Table 2) indicated that no mutations occurred in the lysis gene *3.5*, it may be speculated that the missense mutations in genes *16* and *19.5* could be contributing to the adaptation of T7-△holin phage during the *E. coli* BL21 host infection process.

**TABLE 2:** Mutations arising from T7-△holin during serial passage of infected *Escherichia coli* BL21 cells.

**Gene**	**Function**	**T7-△holin-F1**	**T7-△holin-F10**	**T7-△holin-F20**
1	RNA polymerase	3616 C→T L872F	3125 C→T A708A 3616 C→T L872F	3125 C→T A708A 3616 C→T L872F
1.3	DNA ligase	4762 C→T L152L	4762 C→T L152L	4762 C→T L152L
1.4	Unnamed protein	del 5535–5538 del V	del 5535–5538 del V	del 5535–5538 del V 5543 C→A A35D
1.6	Unnamed protein	5947 C→T T70T	5947 C→T T70T	5947 C→T T70T
1.7	Unnamed protein	6255 G→A K86K	6255 G→A K86K	6255 G→A K86K
2	Inhibits *E. coli* RNAP	6768 T→C N13N	6768 T→C N13N	6768 T→C N13N
2.8	Unnamed protein	8006 G→A E106E	8006 G→A E106E	8006 G→A E106E
5	DNA polymerase	13877 C→T D628D	13877 C→T D628D	13877 C→T D628D
5.3	Unnamed protein	14106 G→A V49I	13963 T→G M1R 14106 G→A V49I	13963 T→G M1R 14106 G→A V49I
5.5	Binds host nucleoid protein H-NS	14399 A→G I24V	14399 A→G I24V	14399 A→G I24V
6.3	Unnamed protein	15901 G→A G10D	15901 G→A G10D	15901 G→A G10D
7	Host range	16627 G→A V7M	16627 G→A V7M	16627 G→A V7M
7.7	Unnamed protein	17355 G→A G10D	17355 G→A G10D	17355 G→A G10D
10A	Major capsid protein		21476 C→A S344stop	21476 C→A S344stop
12	Tail protein	24556 G→A G780R		
15	Internal virion protein	27258 C→T D519D	27258 C→T D519D	27258 C→T D519D
16	Internal virion protein	30656 T→G A895A	30656 T→G A895A	30656 T→G A895A 30666 G→A V899I
19	DNA mature protein	35991 G→A Q415Q	35991 G→A Q415Q	35991 G→A Q415Q
19.5	Unnamed protein	36836 C→A A24D	36836 C→A A24D	36836 C→A A24D 36878 A→G E38G

*Nucleotide number are those of T7 select 415-1b (T7-wt), regardless of their actual location in the original phage.*

### The Optimal Culture Process of T7-△holin

In order to obtain an increased production of progeny phages, the most appropriate values of initial concentration of host bacteria and phages were optimized. Four phage stocks (T7-wt and T7-△holin-F1, F10, F20) were prepared and adjusted to the same titers, and *E. coli* BL21 was infected with varying MOI (=0.01, 0.001, and 0.0001) at a bacterial concentration of *OD*_600 nm_ of 0.5, 1.0, and 1.5, respectively. Phage titers were evaluated at the end of cultivation and are shown in Table 3.

**TABLE 3:** Optimization of cultivation process of T7-△holin-infected *Escherichia coli* BL21 cells.

**Phages**	**MOI**	**Biological titer (PFU/mL)**		
		**Host Density (OD_600 *nm*_ = 0.5)**	**Host Density (OD_600 *nm*_ = 1.0)**	**Host Density (OD_600 *nm*_ = 1.5)**
T7-wt	0.01	4.43E + 09	2.21E + 10	3.40E + 10
	0.001	6.12E + 09	2.93E + 10	4.72E + 10
	0.0001	8.63E + 09	4.30E + 10	5.63E + 10
T7-△holin-F1	0.01	8.67E + 09	6.97E + 09	4.21E + 10
	0.001	4.83E + 09	8.23E + 09	2.26E + 10
	0.0001	2.33E + 10	1.24E + 10	8.41E + 09
T7-△holin-F10	0.01	1.07E + 10	1.27E + 10	3.56E + 10
	0.001	1.33E + 10	2.73E + 10	5.32E + 10
	0.0001	3.27E + 10	5.57E + 10	3.47E + 10
T7-△holin-F20	0.01	1.73E + 10	2.74E + 10	3.68E + 10
	0.001	3.83E + 10	4.90E + 10	5.67E + 10
	0.0001	4.57E + 10	7.38E + 10	5.24E + 10

*T7-△holin-F1, T7-△holin-F10, T7-△holin-F20 stands for the 1st, 10th, and 20th generation of T7-△holin phage, respectively.*

**FIGURE 6:**
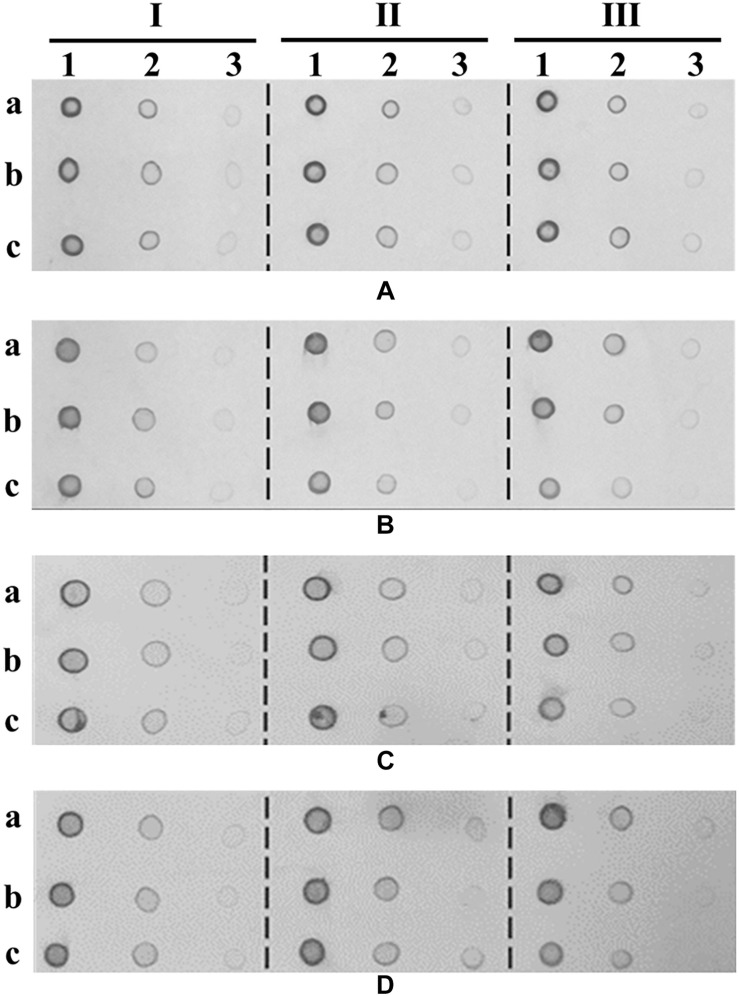
Dot-ELISA assay with T7-Tag^®^ monoclonal antibody. Phage particles recycled after optimization of cultivation process were detected by Dot-ELISA with T7-Tag^®^ monoclonal antibody. **(A)** T7-wt cultivation. **(B)** T7-△holin-F1 cultivation. **(C)** T7-△holin-F10 cultivation. **(D)** T7-△holin-F20 cultivation. Columns I, II, and III represent the initial host cell density of *OD*_600 nm_ = 0.5, 1.0, and 1.5, respectively. Columns 1, 2, and 3 represent the 10-, 100-, and 1,000-fold dilution of each phage sample, respectively. Rows a, b, and c represent the multiplicity of infection values of 0.01, 0.001, and 0.0001, respectively.

The progeny phage titer had a positive relation with the initial host bacteria density when it was infected with a high MOI (0.01) and a middle MOI (0.001). However, when a low MOI (0.0001) was used to infect host bacteria, an initial high density of bacteria (*OD*_600 *nm*_ = 1.5) produced a lower titer of progeny phages compared to a middle density of bacteria (*OD*_600 *nm*_ = 1.0). Host bacteria grown to *OD*_600 nm_ of 1.5 and infected with T7-wt at an MOI value of 0.0001 produced the highest progeny titers (5.63 × 10^10^ PFU/ml). By contrast, host bacteria grown to *OD*_600 nm_ of 1.0 and infected with T7-△holin-F20 at an MOI value of 0.001 produced the highest progeny titers (7.38 × 10^10^ PFU/ml). Thus, through the culture condition optimization, T7-△holin could only produce 1.3 times the number of progeny phage particles compared to T7-wt. Furthermore, when the capsid protein of phage was detected by Dot-ELISA, no significant difference of capsid content was observed (Figures 6A–D) which confirmed that the goal of high titer culture of T7-△holin could not be achieved by process optimization.

## Discussion

T7 phage genes are named as a number and are usually non-essential if the gene includes a decimal (Heineman et al., 2009). Gene *17.5* is the holin of T7 phage, and it creates sudden membrane lesions in a concentration-dependent manner in the phage lysis pathway (Vukov et al., 2000; Wang et al., 2003; Ryan and Rutenberg, 2007). This saltatory manner of holin activity plays a key role in ensuring that production of phage progeny continues unabatedly until the initiation of the lysis program. A previous study identified that lysis was delayed in liquid cultures of a gene *17.5* nonsense mutant of T3 phage (Miyazaki et al., 1978). Moreover, another study has indicated that T7 phage may contain additional holin, since the lack of gene *17.5* did not prevent the evolution to more rapid lysis (Heineman and Bull, 2007). Although the construction of T7-△holin mutant has been reported previously (Heineman et al., 2009), this study is the first to address the molecular evolution of T7-△holin phage during the serial passage process. The direct effects of holin deletion from the T7 phage genome are a delay in the onset of lysis accompanied by an increased burst size (Figure 3 and Table 1). Burst size appeared to be associated with cellular levels of holin protein for the *E. coli* BL-holin host infected with the T7-△holin phage. The induced holin protein accelerated lysis and recovered the burst size back to normal lysis levels (Table 1). These data are consistent with previous reports (Singh and Dennehy, 2014) that the holin protein is a regulator of onset of lysis and lysis delay can enlarge the progeny phages.

In canonical lysis, holin protein accumulates harmlessly in the cytoplasmic membrane until triggering at a specific time to form micron-scale holes and then allowing the soluble endolysin to escape and to degrade the peptidoglycan (Young, 2014). However, it has been reported that when holin is blocked, efficient lysis can still evolve but does not display a recovery that closely approaches the characteristic of normal lysis (Heineman et al., 2009). The host bacterial membrane lesion and internal phage particle accumulation post T7-wt and T7-△holin infection are contrasted by SEM and TEM observation (Figure 2). As expected, the lysis process of T7-wt was consistent with previous reports (Young, 2014; Cahill and Young, 2019), presenting with membrane gap, inner and outer membrane fusion, and release of cytoplasmic contents (Figures 2B,E). By comparison, host bacteria infected with T7-△holin demonstrated a swelling at the pole (Figure 2C) and enlargement of the interval between cell wall and membrane can be observed (Figure 2F). Unfortunately, the mechanism of this phenomenon cannot be explained currently; however, there might be an as-yet unknown means of endolysin transmembrane transportation and subsequent degradation of peptidoglycan. Degradation of peptidoglycan leads to the relaxation of periplasm structure and imbalance of internal osmotic pressure of the cell, eventually resulting in enlargement of the interval (Krupovic et al., 2008; Dewey et al., 2010). In addition, T7-△holin infection should increase the progeny phage particle accumulation in the cytosol due to lysis delay, but phage particles were not clearly visible (Figure 2F) as the phage particles may be concealed by the black background due to incomplete decolorization of the sample slice.

The rate of plaque size enlargement can serve as an index for how efficiently a phage develops and produces progeny within an infected host bacterium (Tabib-Salazar et al., 2018). T7 phage plaques have been reported to enlarge continually in mature *E. coli* lawns (Yin, 1993), which suggested that the T7 phage has evolved specific mechanisms to infect and develop in the stationary phase of host cell growth. Thus, the plaque size formed by the T7-wt and different generations of T7-△holin was compared, as it indirectly reflected the process of evolution of the T7-△holin mutant. As shown in Figure 4, much bigger plaques were formed by T7-△holin-F20 than T7-△holin-F1, which indicates a more rapid infection of host bacteria and more rapid lysis. Onset of lysis and burst size are two parameters that are normally regulated in a trade-off manner to maximize phage fitness (Abedon et al., 2003). It was observed that the plaque size (Figure 4) and the burst size (Table 1) corresponded to each other, and these observations further suggest that small bursts are compensated for by rapid lysis, while delayed lysis usually produces more progeny (Abedon et al., 2003; Wang, 2006; Heineman and Bull, 2007). Burst size, which represents the number of progeny particles a phage produces after one cycle of infection, is not the same as the final production of phage particles in liquid culture. As indicated in Tables 1, 3, T7-△holin-F1 has an enlarged burst size and a delayed lysis onset. Incomplete lysis of host cells is observed in conditions of low MOI and high initial host density, leading to relatively small numbers of progeny. A high-titer culture of T7-△holin cannot be realized merely by optimizing the MOI and initial host density, instead comprehensive parameter optimization and innovative culture process are needed. Furthermore, the mRNA level of gene *10* was stably maintained both in T7-wt and the 1st, 10th, and 20th generations of T7-△holin (Figure 5), which indicates a relatively constant expression of capsid protein and subsequent release of progeny phage particles. By contrast, there was a significant difference in burst size between T7-wt and T7-△holin (Table 1), indicating that the enlarged burst size may be due to increased time for packaging of infective phage particles rather than upregulated expression of capsid protein.

The revised “three-step” model for lysis involves three functional types of protein: holin, endolysin, and spanin (Rajaure et al., 2015). These proteins act sequentially on the inner membrane, peptidoglycan, and outer membrane components of the cell envelope to effect lysis. For T7 phage, the overlapping genes *18.5* and *18.7* apparently encode spanins, which span the periplasm and are involved in disrupting the outer cell membrane under high salt concentration, and they are homologous to the overlapping genes Rz and Rz1 of lambda phage (Summer et al., 2007). In addition, three other T7 genes, i.e., genes *19.2*, *19.3*, and *19.5* encode hypothetical proteins with potential lysis function and are conserved among close relatives of T7 phage (Nguyen and Kang, 2014). If these hypothetical genes are expressed as proteins, they could act as candidates for the unknown lysis-associated factors, especially when the holin gene is deleted, as a new lysis pathway is needed to compensate for inefficient function. Therefore, the mRNA levels of candidate genes of T7-△holin post-infection were measured (Figure 5). The mRNA transcription level of gene *3.5* was significantly upregulated during serial passage, especially at the 20th generation. Gene *3.5* is expressed along with replication genes rather than late genes, it also binds T7 RNA polymerase and inhibits transcription, and it stimulates replication and package of T7 DNA. Thus, it may be speculated that the upregulation of gene *3.5* transcription (Figure 5) may contribute to the lysis onset acceleration and burst size shrinkage of T7-△holin-F20 (Table 1). However, WGS data show that gene *3.5* maintained its genetic stability and no mutations were observed after serial passage (Table 2), which indicates that there was no direct connection between the upregulation and nucleotide sequence change. In contrast, the mRNA level of gene *19.5* increased gradually from the 1st to the 20th generation (Figure 5), with mutation accumulation during the serial passage process (Table 2). Two missense mutations were discovered in gene *19.5*; coincidentally, the mutation in gene *19.5* (36878A→G E38G) was consistent with the report by Heineman et al. (2009) when the evolvability of lysis was studied in T7 phage. Since gene *19.5* has been identified as a gene with a possible lytic function, the identification of the E38G mutation confirms that it is a crucial residue for the compensatory lytic activity of gene *19.5*.

In summary, deletion of the T7 phage holin gene resulted in lysis delay, which allowed the phage more time to assemble a greater number of progeny particles. However, the delay in lysis onset and burst size enlargement of T7-△holin gradually recovered to the normal lysis levels following serial passage of the T7-△holin mutant. Gene *19.5* may be a lysis-associated gene, since mRNA levels were upregulated and two missense mutations had accumulated by the 20th generation. This might result in compensation of inefficient lysis function due to holin deletion. This study shows a new level of adaptation related to the regulation of transcription of genes *16* and *19.5* involved in lysis (E38G mutation in gene *19.5*). It will be worthwhile to further characterize the gene function of gene *19.5* and conduct more detailed microscopic analysis of the T7-wt phage vs. T7-△holin mutants.

## Data Availability Statement

The data presented in the study are deposited in the GenBank repository, accession numbers MZ318361, MZ318362, and MZ318363.

## Author Contributions

HX participated in most of the experiments and drafted the manuscript. XB carried out the rescue of T7-△holin phage and biological characteristic analysis. WH optimized the culture process of T7-△holin phage. KW performed the detection of mRNA level. AW performed the whole-genome sequencing analysis. HD carried out the serial passage of T7-△holin phage. RG, JH, BD, and HC participated in the conception, drafting, and/or editing of the manuscript.

## Conflict of Interest

The authors declare that the research was conducted in the absence of any commercial or financial relationships that could be construed as a potential conflict of interest.

## Publisher’s Note

All claims expressed in this article are solely those of the authors and do not necessarily represent those of their affiliated organizations, or those of the publisher, the editors and the reviewers. Any product that may be evaluated in this article, or claim that may be made by its manufacturer, is not guaranteed or endorsed by the publisher.
