# Supplementary material for: Biological Characterization and Evolution of Bacteriophage T7-△holin During the Serial Passage Process
Source: Front Microbiol. 2021 Aug 2;12:705310. doi: 10.3389/fmicb.2021.705310 (PMC8365609; doi:10.3389/fmicb.2021.705310)
Supplement: Supplementary file 1 [file Data_Sheet_1.docx]

Supplementary material for

**Biological characterization and evolution of bacteriophage T7-△holin during the serial passage process**

Hai Xu ^1,2,3^, Xi Bao^2^, Weiming Hong^1^, Anping Wang^1^, Kaimin Wang^4^, Hongyan Dong^1^, Jibo Hou^2^, Roshini Govinden ^3^, Bihua Deng^2*^, Hafizah Y. Chenia^3^[[1]](#footnote-2)^*^

*^1^**Jiangsu Key Laboratory for High-Tech Research and Development of Veterinary Biopharmaceuticals, J**iangsu Agri-animal Husbandry Vocational College, Taizhou, 225300, Jiangsu province, PR China*

*^2^* *Institute of Veterinary Immunology &Engineering, Jiangsu Academy of Agricultural Science, Nanjing 210014, Jiangsu Province, PR China*

*^3^**School of Life Sciences College of Agriculture, Engineering and Science, University of KwaZulu-Natal, Durban, 4001, South Africa*

*^4^ Animal, Plant and Food Test Center of Nanjing Customs, Nanjing 210095, Jiangsu province, PR China*

This file includes:

Table 1S

Figure 1S

Figure 2S

| Table S1: PCR primers used in mutant phage construction, identification, and phage mRNA quantification. | | |
| --- | --- | --- |
| Name | Sequence (5^’^→3^’^) | Function |
| F1 | ATAGGCCGTTGTGGCCACTGATGGT | Amplification from*SfiI*site to T7-wt genomic position 33720. |
| R1 | AGAGCATGCCTTGTACCTCCTTGAGAGTCC |  |
| F2 | GCCGCATGCTATGTATGGAAAAGGATAAGA | Amplification from T7-wt genomic position 33925 to the right terminal position 37314. |
| R2 | AGGGACACAGAGAGACACTCAAGGT |  |
| F3 | TAGTGCTGGCGGTGGGGTA | Identification of gene *17.5* deletion phage. |
| R3 | CCTTGAGTATATCACTGTAA |  |
| F3.5 | GGTTCTCCAACGTCTTATGGA | RT-qPCR detection of gene *3.5*. |
| R3.5 | AACGTGTGTCCGTCAGAGAAC |  |
| F10a | CGACTCACTATAGGGAGACCA | RT-qPCR detection of gene *10a*. |
| R10a | TGAGCGCATATAGTTCCTCCTT |  |
| F16 | CCAAGTGATTATGATGGTCTG | RT-qPCR detection of gene *16*. |
| R16 | CTCCCTCAAGTTAACACCGTT |  |
| F17 | CACTTCCACGTAGGTCAGGC | RT-qPCR detection of gene *17*. |
| R17 | AATCCAGATATTGCGGAAGC |  |
| F18.5 | CTCAAGGCCACTACAGATAGT | RT-qPCR detection of gene *18.5*. |
| R18.5 | AACGCCACGAAGTCTCCTTTC |  |
| F19.5 | GTTCCGCTTATTGTTGAACCT | RT-qPCR detection of gene *19.5*. |
| R19.5 | CTAATCGCTACAAGTGAGTA |  |
| F16S | GCAGAAGAAGCACCGGCTAA | RT-qPCR detection of *Escherichia coli* BL21 16S mRNA. |
| R16S | GGTATTCCTCCAGATCTCTA |  |
| Underline represents restriction enzyme site. | | |


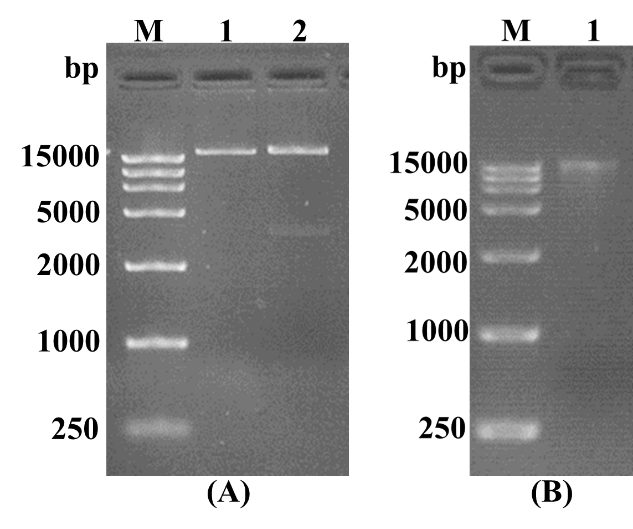


Figure S1: (A). Purification and digestion analysis of T7 phage genome. M, DL15000 marker. 1, T7-wt phage genome. 2, *Sfi*Ⅰdigestion of T7-wt genome. (B). Gel extraction of gene fragment. M, DL 15000 marker. 1, Gel extraction of the upstream part of *Sfi*Ⅰ site.


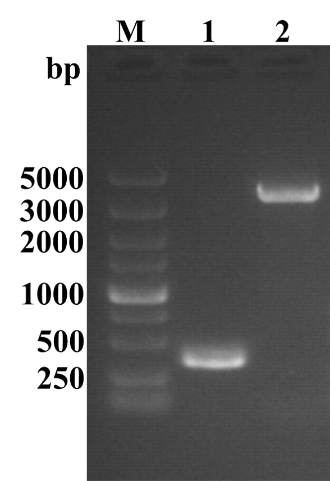


Figure S2: Preparation of gene fragments of gene*17.5* up and down stream part. M, DL5000 marker. 1, Gene fragment from *Sfi*Isite to T7-wt genomic position 33720. 2, Gene fragment from T7-wt genomic position 33925 to the right terminal position 37314.

1. *Corresponding authors at School of Life Sciences College of Agriculture, Engineering and Science, University of KwaZulu-Natal, Durban, South Africa and Institute of Veterinary Immunology &Engineering, Jiangsu Academy of Agricultural Science, Nanjing 210014, Jiangsu Province, PR China. They contributed equally to the work.

   Tel: +27 31 260 8281, +86 25 83392068; Fax: +27 31 260 7809, +862584392028

   E-mail address:[cheniah@ukzn.ac.za](mailto:cheniah@ukzn.ac.za), dengbihua1981@163.com [↑](#footnote-ref-2)
